# Bortezomib (PS-341) Treatment Decreases Inflammation and Partially Rescues the Expression of the Dystrophin-Glycoprotein Complex in GRMD Dogs

**DOI:** 10.1371/journal.pone.0061367

**Published:** 2013-04-08

**Authors:** Karla P. C. Araujo, Gloria Bonuccelli, Caio N. Duarte, Thais P. Gaiad, Dayson F. Moreira, David Feder, José E. Belizario, Maria A. Miglino, Michael P. Lisanti, Carlos E. Ambrosio

**Affiliations:** 1 Department of Surgery, School of Veterinary Medicine and Animal Science, University of São Paulo, São Paulo, SP, Brazil; 2 Department of Stem Cell Biology and Regenerative Medicine and Cancer Biology, Thomas Jefferson University, Philadelphia, Pennsylvania, United States of America; 3 Department of Physiotherapy, Faculty of Biological Science and Health, UFVJM, Diamantina, MG, Brazil; 4 Department of Pharmacology, Institute of Biomedical Science, University of São Paulo, São Paulo, SP, Brazil; 5 Department of Pharmacology, ABC School of Medicine, Santo Andre, SP, Brazil; 6 Laboratory for Orthopaedic Pathophysiology and Regenerative Medicine, Istituto Ortopedico Rizzoli, Bologna, Italy; 7 Breakthrough Breast Cancer Research Unit, Institute of Cancer Sciences, University of Manchester, Manchester, United Kingdom; 8 Department of Veterinary Medicine, Faculty of Animal Sciences and Food Engineering, University of São Paulo, Pirassununga, SP, Brazil; Brigham and Women's Hospital, Harvard Medical School, United States of America

## Abstract

Golden retriever muscular dystrophy (GRMD) is a genetic myopathy corresponding to Duchenne muscular dystrophy (DMD) in humans. Muscle atrophy is known to be associated with degradation of the dystrophin-glycoprotein complex (DGC) via the ubiquitin-proteasome pathway. In the present study, we investigated the effect of bortezomib treatment on the muscle fibers of GRMD dogs. Five GRMD dogs were examined; two were treated (TD- Treated dogs) with the proteasome inhibitor bortezomib, and three were control dogs (CD). Dogs were treated with bortezomib using the same treatment regimen used for multiple myeloma. Pharmacodynamics were evaluated by measuring the inhibition of 20S proteasome activity in whole blood after treatment and comparing it to that in CD. We performed immunohistochemical studies on muscle biopsy specimens to evaluate the rescue of dystrophin and dystrophin-associated proteins in the muscles of GRMD dogs treated with bortezomib. Skeletal tissue from TD had lower levels of connective tissue deposition and inflammatory cell infiltration than CD as determined by histology, collagen morphometry and ultrastructural analysis. The CD showed higher expression of phospho-NFκB and TGF-β1, suggesting a more pronounced activation of anti-apoptotic factors and inflammatory molecules and greater connective tissue deposition, respectively. Immunohistochemical analysis demonstrated that dystrophin was not present in the sarcoplasmic membrane of either group. However, bortezomib-TD showed higher expression of α- and β-dystroglycan, indicating an improved disease histopathology phenotype. Significant inhibition of 20S proteasome activity was observed 1 hour after bortezomib administration in the last cycle when the dose was higher. Proteasome inhibitors may thus improve the appearance of GRMD muscle fibers, lessen connective tissue deposition and reduce the infiltration of inflammatory cells. In addition, proteasome inhibitors may rescue some dystrophin-associated proteins in the muscle fiber membrane.

## Introduction

Golden retriever muscular dystrophy (GRMD) is a degenerative myopathy corresponding to Duchenne muscular dystrophy (DMD) in humans. Both GRMD and DMD are caused by the absence of a functional dystrophin protein. In GRMD, this absence is the result of a frame-shifting point mutation in the dystrophin gene, whereas deletions are the most frequent mutations in DMD patients [1],[2].

Similarly to DMD patients, GRMD dogs suffer from repeated cycles of muscle necrosis and regeneration, muscle wasting and fibrosis, postural abnormalities, respiratory or heart failure and premature death [3],[4],[5],[6]. As GRMD dogs closely resemble DMD patients, both in terms of body weight and in the pathological expression of the disease [7], they are excellent animal models for the study of pathogenic mechanisms and therapeutic interventions.

Dystrophin is located beneath the sarcolemma and is part of a large dystrophin-dystroglycan complex termed the dystrophin-glycoprotein complex (DGC); it includes the dystroglycan complex (α and β) and the sarcoglycan complex (α, β, γ and δ) [8]. The DGC is a critical link in the transmission of force between the contractile machinery of muscle fibers and the extracellular matrix. When dystrophin is defective or absent, the myofiber is fragile and the sarcolemma is readily damaged in response to exercise, leading to myofiber necrosis [1], [4]. The loss of dystrophin leads to the absence of or a great reduction in the components of the DGC, as has been described for skeletal muscle fibers from DMD patients and mdx mice [9], [10].

The current treatment for DMD is the administration of corticosteroids; these broad-based anti-inflammatory drugs decrease inflammatory cell populations in dystrophic muscle and increase myofiber mass, although their precise mechanism of action in DMD is not yet known and is under intense investigation [11],[12]. Steroids are associated with severe adverse side effects such as weight gain and osteoporosis, and the response to steroid therapy is variable among individual patients [13], [14].

Bortezomib (Velcade®) is a dipeptide boronic acid proteasome inhibitor that works by reversible inhibition of the chymotrypsin-like activity of the proteasome [15], [16]. Previous reports have shown that proteasome inhibitors are able to block the activation of nuclear factor-κB (NFκB). This factor is involved in inflammatory and acute stress responses. Studies have reported that the NFκB pathway is activated in DMD and that it is involved in muscle degeneration and regeneration in dystrophin-deficient fibers [17], [18]. Treatment with bortezomib and another proteasome inhibitor, MLN-273, caused a significant decrease in the expression of the activated form of NFκB in the skeletal muscle of mdx mice [19].

Other mechanisms involved in DMD are related to transforming growth factor-beta 1 (TGF-β1), which is the best-characterized fibrogenic mediator [20]. The activation of NFκB and the acute activation of TGF-β1 in human dystrophin-deficient muscle tissue cause the failure of metabolic pathways later in the disease and appear to be associated with symptoms and muscle wasting in DMD [21]. TGF-β1 is overexpressed in human dystrophic muscle, in degenerative muscle disease [22],[23] and in the skeletal muscle of dogs with GRMD [24]. Increased TGF-β1 mRNA levels are also associated with the initial stage of tissue fibrosis [22]. These findings suggest that TGF-β1 is involved in the fibrotic process of human muscle dystrophy.

Several lines of evidence have suggested that enhanced activation of the proteasome pathway underlies the pathogenesis of various diseases including skeletal muscle atrophy and muscle dystrophy [25],[26],[27],[28],[29]. This has led to the suggestion that proteasome inhibitors such as bortezomib might rescue the structure of dystrophin and improve muscle condition.

Recent *in vitro* studies on Duchenne and Becker muscular dystrophy and *in vivo* studies in mdx mice have demonstrated that inhibitors of the proteasome pathway can effectively block the degradation of dystrophin and dystrophin-associated proteins and improve the morphology of dystrophin-deficient skeletal muscle [19], [27], [30].

It should be noted that the use of GRMD dogs for collecting data related to the reconstruction of muscle function is not trivial [7]. Raising just 2 GRMD dogs and treating them with drugs such as bortezomib for one month costs approximately US$ 15,000.00. We therefore agree with the recent article [31] about the limitations of using GRMD dogs during preclinical trials.

The present study analyzed the effect of bortezomib treatment on the muscle fibers of GRMD dogs. Dogs were treated with bortezomib using the same treatment regimen used for multiple myeloma and were assessed for activation of the ubiquitin-proteasome proteolytic pathway during the rapid loss of muscle protein in dystrophin-deficient skeletal muscle. Pharmacodynamics were evaluated by measuring the inhibition of 20S proteasome activity in whole blood following treatment and comparing it to that of untreated dogs (CD). We performed immunohistochemical analysis of muscle biopsy specimens to evaluate the rescue of dystrophin and dystrophin-associated proteins in the muscles of GRMD dogs treated with bortezomib.

## Methods and Ethics Statement

Five GRMD dogs were selected from the GRMD-Brazil kennel at the Department of Surgery of the University of São Paulo. This research was approved by the Bioethics Committee of the School of Veterinary Medicine and Animal Science, protocol number 893/2006.

### Animal Models

Five GRMD dogs (four months old at the beginning of the study) and three healthy dogs were used to analyze and compare the architecture of muscle fibers. Four of the dogs came from the same litter and one came from another litter, but all were born during the same week. We chose dogs of the same age to reduce phenotypic variability, which is characteristic of the canine model of muscular dystrophy. We followed our previous approach using morphological tools to characterize different phenotypes in GRMD dogs [32].

The dogs were kept under humane conditions that included proper meals, hygiene and monitoring by veterinarians. The diagnosis of muscular dystrophy was confirmed in GRMD dogs by DNA testing at one week of age.

The GRMD dogs were separated into two groups: two treated dogs (TD) and three control dogs (CD). Beginning the study with four-month-old dogs was important because they remained ambulant and because clinical signs of the disease were not severe, as the fibrotic process had not yet fully developed at this age [33].

Skeletal muscle tissue was collected from TD and CD for further analysis before treatment (time zero, T0) and after treatment (time one, T1) 9 weeks later. A portion of the samples collected in the biopsy was frozen at −80°C until western blot analysis.

### Administration of Bortezomib

A 3.5-mg vial of bortezomib (Velcade®) was reconstituted with 3.5 mL normal saline (USP), such that the reconstituted solution contained 1 mg/mL. Administration of bortezomib was performed with a 3 to 5 second intravenous injection twice weekly (Mondays and Thursdays) for 2 weeks (days 1, 4, 8, and 11), followed by a 10 day rest period (days 12–21). This protocol constituted 1 cycle of treatment in accordance with the therapy stipulated by the manufacturer. A total of 3 cycles of treatment were administered.

The doses in this study were in accord with those administered to human myeloma patients [34]. The first cycle was 1.3 mg/m^2^, the second was 1.45 mg/m^2^ and the third cycle was 1.65 mg/m^2^. We changed the dose between cycles because the effect of the proteasome inhibitor is dose-dependent and because better results have been observed at higher concentrations [19], [27]. The dosage was partially influenced by pharmacodynamic data that predict a gradual approach up to 90% inhibition as the target for the maximal level of safe inhibition [35].

### Histological and Morphometric analyses

Muscle samples were fixed in a 4% paraformaldehyde solution. Muscle sections of 5 µm thickness were stained with hematoxylin and eosin to evaluate morphology and disease progression. Transverse sections were stained with picrosirius red to distinguish skeletal muscle from collagen fibers. To accurately quantify collagen-positive areas, thirty randomly selected low-power fields per muscle section slide from each dog at each time point (T0 and T1) were microscopically analyzed (Axioplan 2; Carl Zeiss, Inc.). Digital pictures were captured with a video archival system using a digital television camera system (AxioCam High Resolution Color, Carl Zeiss, Inc.). An automated software analysis program (KS400, Carl Zeiss, Inc.) was used to determine the percentage of stained areas in the digital photomicrographs [36].

### Immunohistochemical analysis

The paraffinized 5 µm sections of skeletal muscle were placed in an incubator at 55°C for 1 hour, deparaffinized and dehydrated, boiled in citrate buffer and incubated with 3% hydrogen peroxide in phosphate-buffered saline (PBS, Biochrom KG, Germany) to inhibit endogenous peroxidases. The sections were then washed two times in PBS and pre-treated with 10% normal goat serum diluted in PBS to block non-specific binding. The antibodies were diluted in 10% normal goat serum. Incubations were conducted overnight at 4°C. After three washes in PBS, sections were incubated with a biotinylated secondary goat anti-mouse IgG antibody or a goat anti-rabbit IgG antibody (Vector®) diluted 1∶500 in PBS. For all antibodies, immunoreactivity was detected by a streptavidin-biotin-peroxidase (Dako®) technique using 3,3′-diaminobenzidine (Dako®). Sections were then stained for 30 s with hematoxylin, dehydrated, and mounted.

Phospho-NFκB-positive nuclei were counted in 10 random fields at 40× magnification. A Student's *t*-test was used to evaluate the results.

Antibodies directed against α-dystroglycan (NCL-α-DG) diluted in PBS 1∶50, β- dystroglycan (NCL-β-DG) at 1∶25 and dystrophin (NCL-DYS1) at 1∶10 were purchased from Novocastra (Newcastle upon Tyne, UK). TGF-β1 was purchased from Santa Cruz, and phospho-NFκB was purchased from Cell Signaling; both were diluted 1∶50 in PBS. The proteasome inhibitor bortezomib (Velcade®) was purchased from Janssen-Cilag São Paulo (Butanta, São Paulo, Brazil).

### Transmission Electron Microscopy (TEM)

Small pieces of muscle were fixed in Karnovsky modified solution (2.5% glutaraldehyde and 2% paraformaldehyde in 0.1 M phosphate buffer). Fixed muscle samples were post-fixed in 1% OsO_4_ and embedded in Epon-Araldite. Semi-thin (1 µm) sections were stained with toluidine blue for detection of the region of choice, and thin sections were stained on the grid with uranyl acetate and lead citrate and examined in a Phillips 268D transmission electron microscope at 60 or 80 kV.

### Western blot analysis

Skeletal muscle tissues were harvested, minced with scissors, homogenized in a Polytron tissue grinder for 30 s at medium speed, and solubilized in buffer containing 10 mM Tris-HCl (pH 8.0), 150 mM NaCl, 5 mM EDTA, 1% Triton X-100, and 60 mM octyl glucoside for 45 min at 4°C. Samples were centrifuged at 13,000× *g* for 10 min at 4°C to remove insoluble debris. The protein concentration was determined using a BCA kit (Pierce). Soluble proteins were resolved by SDS-PAGE (10% acrylamide) and transferred to nitrocellulose membranes. Blots were blocked for 1 hour in TBST (10 mM Tris-HCl, pH 8.0, 150 mM NaCl, 0.2% Tween 20) containing 5% BSA. The membranes were incubated overnight with the appropriate primary antibody diluted in TBST/1% BSA. After three washes with TBST, the blots were incubated for 30 minutes with horseradish peroxidase (HRP)-conjugated secondary antibodies diluted in TBST/1% BSA. Antibody-bound proteins were detected using an ECL detection kit (Pierce). The blots were then analyzed using ImageJ software [37]. See figures S1 and S2, for methodological comprehension.

### Clinical pathology and clinical signs

Blood from each dog was collected once a week for standard serum biochemistry measurements and hematological examination during bortezomib administration. These samples were processed by the Clinical Laboratory of the Department of Small Animals, University of São Paulo. The progression of muscular dystrophy was monitored by clinical examinations performed once per week for eleven weeks, beginning one week before the administration of bortezomib and ending one week after the last dose. The clinical examination included body condition, posture and gait [32], [38], [39], hydration status, body weight, peripheral lymph nodes, cardiac and respiratory auscultation, palpation and abdominal inspection. The trunk and limbs were palpated and inspected. Muscle mass and tone were evaluated [3], [40]. We also considered the phenotypic variability associated with different measurement tools, such as physical features, the passive range of motion and the circumference of the limbs and thorax [39], while evaluating the clinical symptoms.

### Proteasome activity measurement in blood lysates

The chymotryptic activity of proteasomes was assessed using a fluorogenic substrate assay [35], [41]. Venous blood (3 mL) was collected from untreated and bortezomib-treated dystrophic dogs in heparin-containing tubes 1, 4, and 24 hours post-treatment during the first week of each cycle and stored at −20°C until processing. The samples were placed at ambient temperature and resuspended in PBS (1∶1). The tubes were centrifuged at 2,000× *g* for 30 minutes at 4°C. The plasma was removed, and the buffy coat samples were resuspended in 1 mL of PBS, aliquoted into tubes and frozen at −20°C. Briefly, whole blood cells were lysed with 5 mmol/L EDTA (pH 8.0) for 1 hour and centrifuged at 600× *g* for 10 minutes at 4°C. The resultant whole blood lysate samples were added to substrate buffer (20 mmol/L HEPES, 0.5 mmol/L EDTA, 0.05% sodium dodecyl sulfate, and 60 mmol/L chymotrypsin substrate-Suc-Leu-Leu-Val-Tyr–amido-4-methylcoumarin (AMC) (Calbiochem-Novabiochem)). Kinetic measurements were performed at 37°C for one hour by monitoring AMC release from the synthetic peptide substrate LLVY-AMC. The samples from each animal were tested at least three times for each time point, and the average was calculated. The inhibition percentage was calculated using the expression 100 (1SpAI/SpAu), where SpAI is the chymotryptic activity of the proteasome in the presence of the inhibitor bortezomib and SpAu is the chymotryptic activity in the absence of bortezomib.

### Statistical analysis

Non-parametric tests were used because the sample size was relatively small. A Wilcoxon test was used to compare the percentage of connective tissue deposition between the TD and CD groups. Proteasome inhibition was analyzed in the samples using a 95% confidence interval. Both of these tests were assessed by the Core Team program (2008), and *P* values<0.05 were considered statistically significant. For phospho-NF-κB, the number of positive nuclei per field was quantified. The results are expressed as the mean ± SEM for the CD and TD groups (n = 2). Statistical significance was set at P<0.05 using Student's *t*-test.

## Results

### Histology and ultrastructural analysis

At the first (T0) biopsy, which was made at three months of age and before bortezomib therapy, the muscle fibers showed myofiber hypertrophy, fiber necrosis and regeneration. Discrete endomysial fibrosis and myofiber atrophy were also observed. At this stage, myofibers showed a basophilic cytoplasm and were designated regenerative fibers.

At T1, at the end of the experiment, small fibers undergoing phagocytosis were observed. The amount of these fibers was increased compared to T0. The inflammatory process was predominantly composed of macrophages. At this time, there was a slight increase in endomysial and perimysial connective tissue (endomysial fibrosis), and marked myofiber atrophy in all sections was observed.

**Figure 1:**
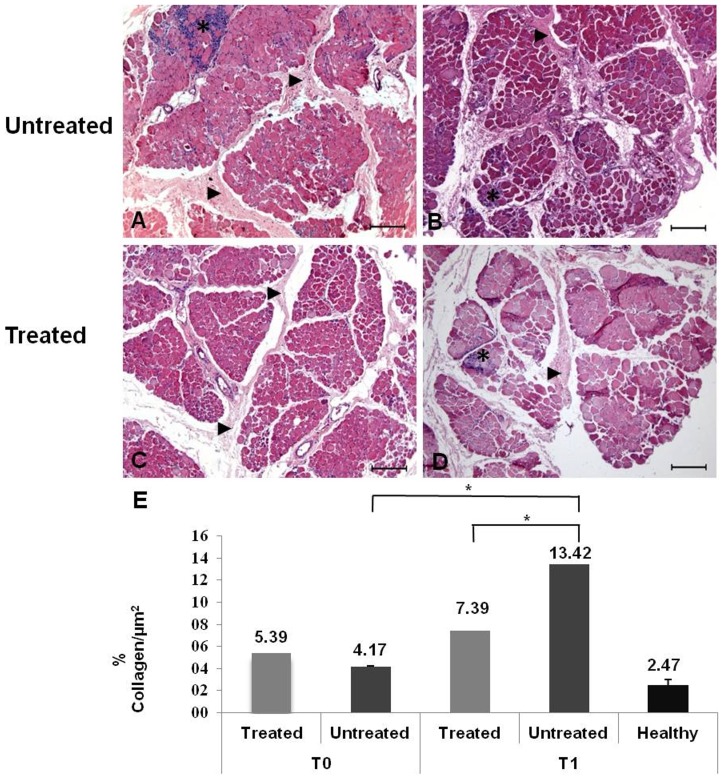
Histological analysis of H&E-stained skeletal muscle fibers after treatment with bortezomib and muscle collagen morphometry. A and B: CD showed a greater deposition of connective tissue in the endomysium and perimysium (▸), and the inflammatory cells formed groups or massive lesions (*) with a poor histopathological appearance. C and D: TD showed lower deposition of connective tissue in endomysium and perimysium (▸) and a lower presence of inflammatory cells (*); Original magnification: 10×; bar: 200 µm. E: Mean and SD of muscle collagen morphometry of slides stained with picrosirius red followed by quantitative analysis. Muscle from healthy dogs and TD and CD before (T0) and after (T1) treatment with bortezomib. The p-value was <0.0001 for comparing the collagen at T0 and T1 for CD. At T1 there was a statistically significant difference between the TD and CD, with higher collagen levels in CD (p = 0.0028).

Skeletal muscle collected at T1 from CD showed an abnormal-affected fiber-forming group of lesions characterized by inflammatory cell infiltration. These dogs presented myopathic changes such as variability in fiber diameter, lymphocytic invasion and increased deposition of connective tissue in the perimysium. Meanwhile, the two TD (K1 and B7) showed a more uniform fiber diameter and less lymphocytic invasion, suggesting an attenuated inflammatory process and reduced signs of muscular dystrophy. The same phenomena were not observed in CD (K4 and K6) (Figures 1A–D), showing that systemic administration of bortezomib seems to ameliorate muscle fiber degeneration, thereby improving skeletal muscle pathology.

At T0, there was no difference between the collagen deposition areas of TD and CD. However, at T1, TD showed 7.39% collagen per µm^2^ and CD showed 13.42% collagen per µm^2^. In 30 random thin-section images from each dog, there was a significant difference between T0 and T1 (p<0.05) for CD, and a significant difference between TD and CD at T1 (p = 0.0028). These data suggest a greater degree of muscle fibrosis in CD muscle (Figure 1E).

**Figure 2:**
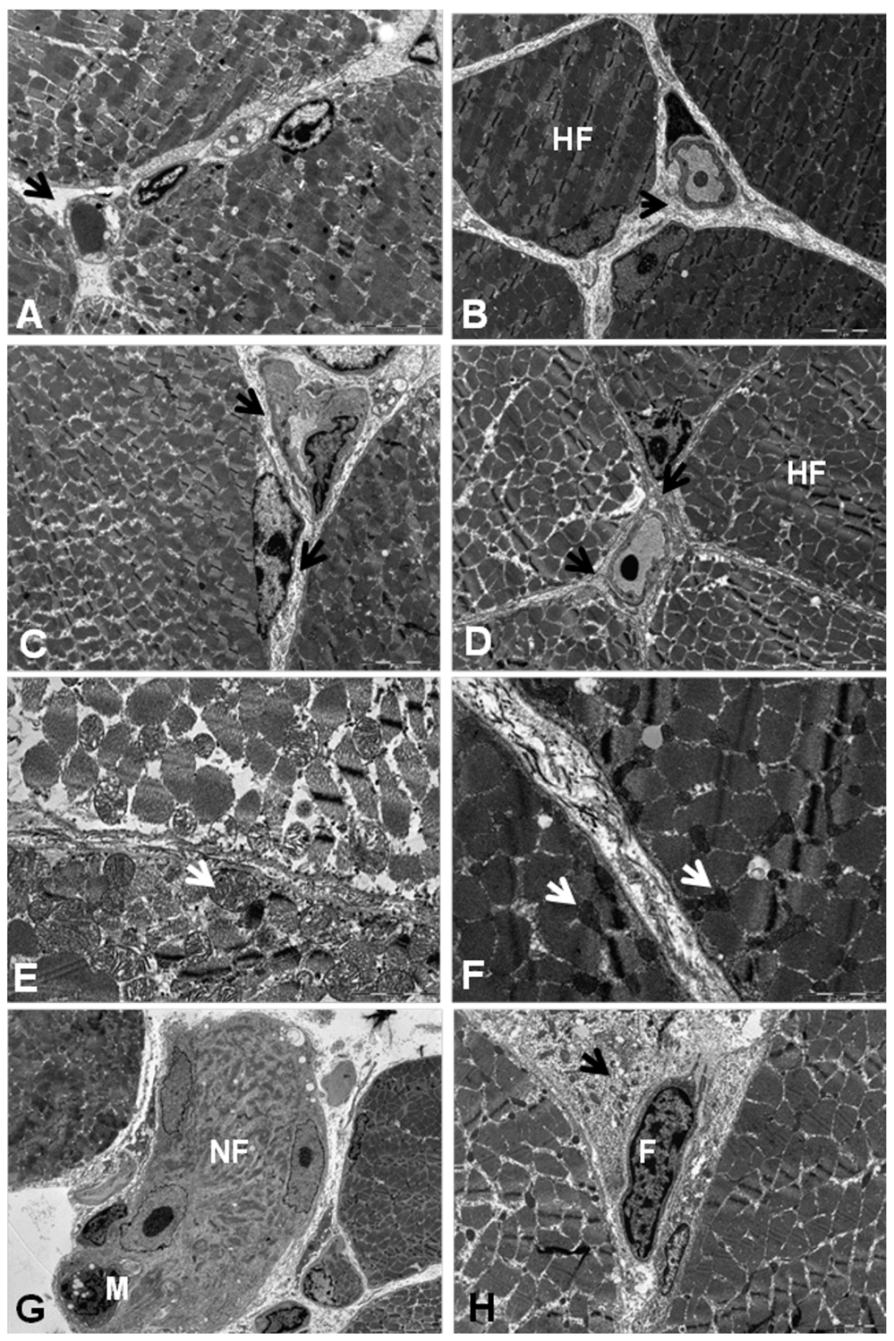
Ultrastructural analysis of connective tissue in muscles from GRMD dogs. **A:** Muscle from a healthy dog. There was a narrow endomysium space and a lower deposition of connective tissue (→). **B:** An untreated GRMD dog (CD). The endomysium of this dog exhibits a higher deposition of connective tissue and hypercontracted fibers (HF). **C and D:** Treated GRMD dogs (TD) showed a lower deposition of connective tissue and endomysium, and few fibers were hypercontracted (HF). Original magnification: 3,500×. **E:** In muscles from healthy dogs, the mitochondria were preserved and had the same electron density as the fibers, and the mitochondrial cristae were visible. **F:** GRMD dogs demonstrated abnormal mitochondria, had a higher electron density, and were smaller, and the cristae were not visible. **G and H:** Abnormal fiber (**NF**) with macrophage invasion (**M**), complete loss of membrane integrity and myofibrillar structure showing a finely granular cytoplasm. Activated fibroblasts (**F**) with a prominent rough endoplasmic reticulum (→) were present in the endomysium. Original magnification: A and B, 8,900×; C, 3,500×; D, 5,600×.

Confirming the findings from muscle histology, transmission electron microscopy (TEM) analysis showed infiltration of inflammatory cells, degenerated fibers, and the presence of activated fibroblasts in the perimysium. The TEM data indicate that connective tissue synthesis and fibrosis were present in all studied dogs; however, the CD showed greater deposition of collagen fibers in the endomysium and perimysium (Figures 2A–D).

Ultrastructural analysis also demonstrated abnormal mitochondria in the cytoplasm and close to the sarcoplasmic membrane in all studied dogs. These mitochondria had a higher electron density and were smaller than those observed in healthy dogs. The morphology of the mitochondria was preserved, and the cristae had a similar electron density to those present at the myofibers of healthy dogs. Abnormal mitochondria were observed in almost all healthy-appearing muscle from the GRMD dogs (Figures 2E and F).

Fully abnormal fibers showed a complete loss of myofibrillar structure and a finely granular cytoplasm associated with loss of membrane integrity. Satellite cells associated with degenerating fibers appeared to be activated, with nuclear enlargement and clearing, reduced heterochromatin, and a greater number of cytoplasmic organelles. Numerous macrophages and fibroblasts with prominent rough endoplasmic reticulum were observed in the endomysium (Figures 2G and H).

### Immunohistochemistry (IHC)

Previous studies have shown that treatment with proteasome inhibitors can block protein degradation and rescue dystrophin and dystrophin-associated proteins *in vivo* in the muscle fibers of mdx mice and *in vitro* in human muscle [19], [27], [30]. Local administration of bortezomib and MLN273 were effective at rescuing the expression and plasma membrane localization of dystrophin and dystrophin-associated proteins α-dystroglycan, β-dystroglycan, and α-sarcoglycan [19].

**Figure 3:**
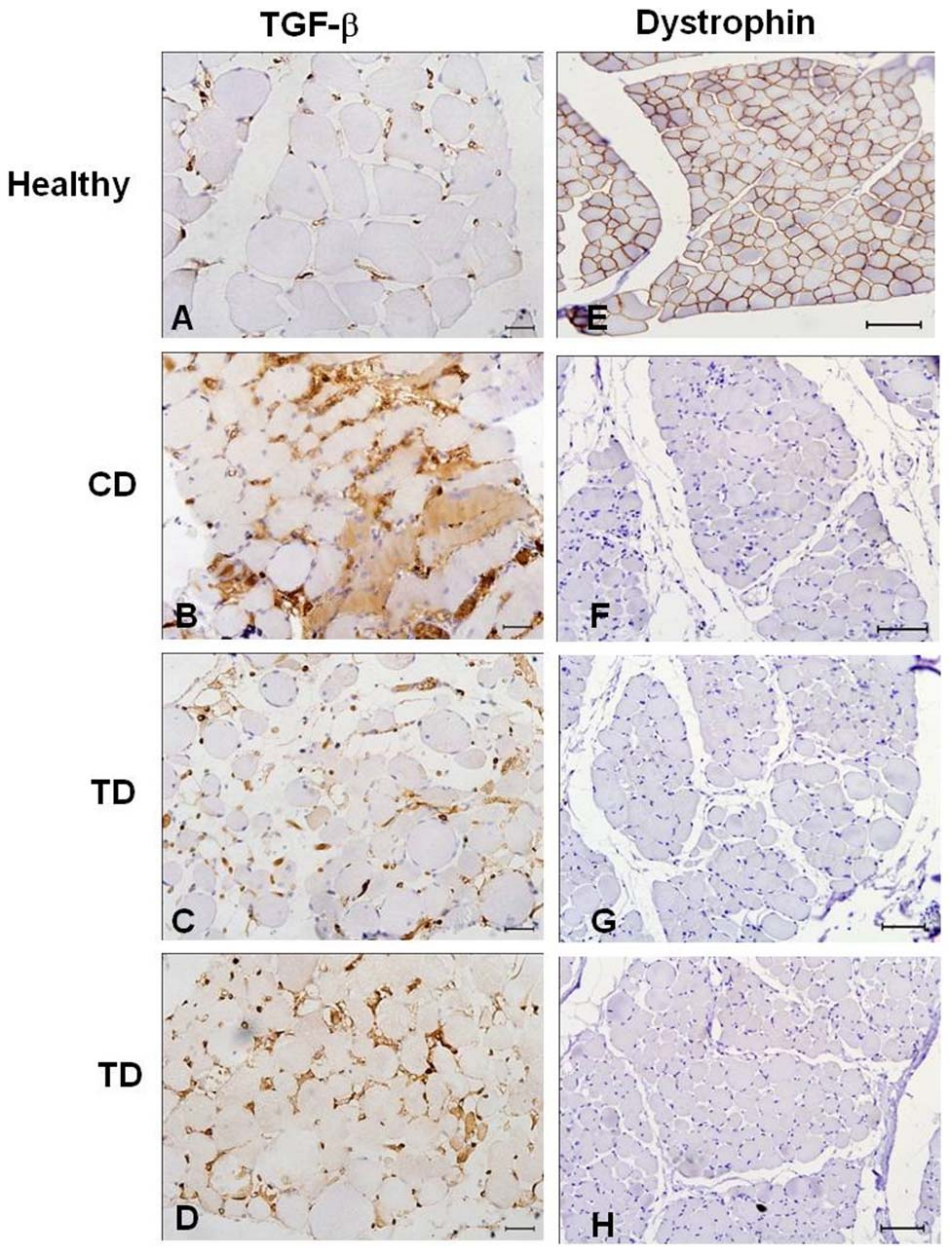
Immunohistochemistry of TGF-β and dystrophin in skeletal muscle. **A and E.** Muscle from healthy dogs. TGF-β is detected around the vessels in muscles (A), α-dystroglycan (E) and dystrophin (I) patterns in the sarcoplasmic membrane. **B:** Muscle from CD at T1 shows greater TGF-β expression in the endomysium of the fibers and more deposition of connective tissue at T1. **C and D:** TD after treatment with bortezomib (T1) showed lower deposition of connective tissue and lower expression of TGF-β in the endomysium of the fibers. Original magnification: 40×; bar: 50 µm. **F, G and H:** Neither untreated (CD) (J) nor treated GRMD dogs (TD) (K and L) showed expression of dystrophin in the sarcoplasmic membrane, indicating that bortezomib did not rescue this protein during the treatment. Original magnification: 20×; bar: 100 µm.

To confirm the results obtained by histology and ultrastructural analysis of connective tissue deposition and fibrosis, we analyzed the expression of TGF-β by immunohistochemistry. The untreated dogs showed higher TGF-β expression in the slides analyzed, indicating higher levels of connective tissue deposition and fibrosis in these animals (Figures 3A–D).

Immunohistochemical staining for the carboxy-terminal portion of dystrophin was positive in the control samples from healthy dogs and negative in muscle sections of GRMD dogs from both groups. The treated dogs did not show dystrophin staining in the cytoplasmic membrane (Figures 3E–H), suggesting that bortezomib treatment is not an effective way to rescue dystrophin production in the muscle membrane.

**Figure 4:**
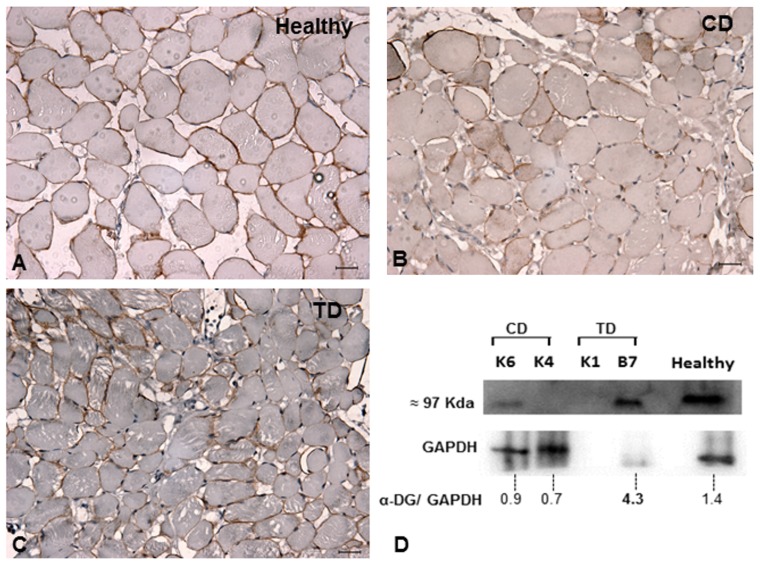
Immunohistochemistry and western blot for α-dystroglycan. **A:** Muscle from a healthy dog shows the α-dystroglycan pattern in sarcoplasmic membranes. **B:** Untreated GRMD dog (CD). **C and D:** Western blot and immunohistochemical analysis from a TD showing higher expression of α-dystroglycan in muscle fibers than CD after treatment with bortezomib. Using ImageJ software and measuring the blot band intensity, we suggest that TD-B7 had a 4-fold increase in the expression of α-dystroglycan. Original magnification: 40×; bar: 50 µm.

**Figure 5:**
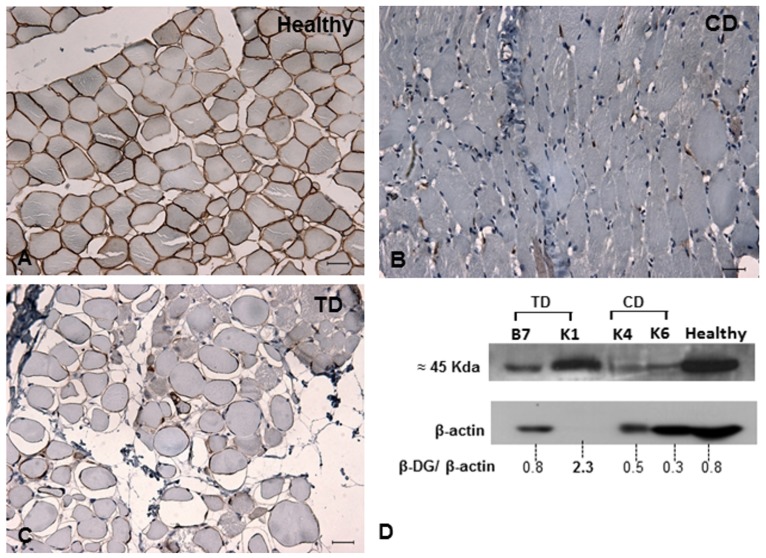
Immunohistochemistry and western blot for β-dystroglycan. **A:** Muscle from a healthy dog shows the β-dystroglycan pattern in sarcoplasmic membranes. **B:** Untreated GRMD dog. **C and D**: Western blot and immunohistochemical analysis from a TD show higher expression of β-dystroglycan in muscle fibers than CD after treatment with bortezomib. Using ImageJ software and measuring the blot band intensity, we found that TD-K1 had a 2-fold increase in the expression of β-dystroglycan. Original magnification: 40×; bar: 50 µm.

**Figure 6:**
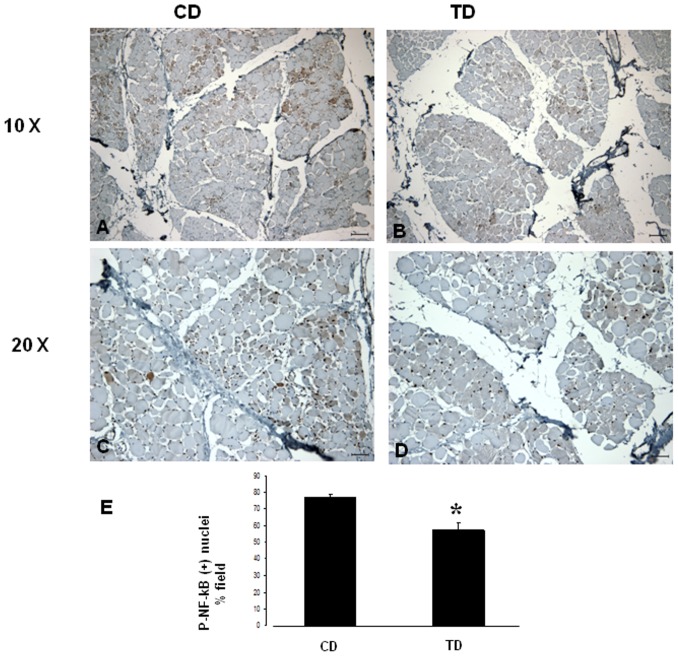
Immunohistochemistry of phospho-NFκB in skeletal muscle. **A and C:** Muscle from a CD shows higher expression of the (active) phospho-NFκB in the nuclei of muscle fibers. **B and D:** GRMD dogs (TD), after treatment with bortezomib, showed a lower expression of phospho-NFκB in the nuclei of fibers, indicating proteasome inhibition and preservation of inactive NFκB in the cytoplasm. Original magnification: A and B, 10×; bar, 200 µm; C and D, 20×; bar, 100 µm. **E:** The phospho-NFκB positive nuclei were counted in 10 random fields, with images captured at 40×. Student's *t*-test was used to evaluate these results (* p<0.05). The CD showed more phospho-NFκB-positive nuclei, indicating more activation of proteasomal activity inducing pro-apoptotic factors and inflammatory molecules.

Among the other proteins in the dystrophin-glycoprotein complex, the expression levels of α-dystroglycan and β-dystroglycan were increased in GRMD dogs treated with bortezomib compared with the untreated dogs (Figures 4 and 5). This result indicates that bortezomib rescues the expression of these proteins and ameliorates the histopathological phenotype. Using ImageJ software and analyzing the expression of the protein of interest divided by the expression of the internal control, we observed a fourfold increase in the expression of α-dystroglycan (TD-B7) and a twofold increase in the expression of β-dystroglycan for TD-K1 (Figure 4D and Figure 5D). However the studies performed suggest a rescue of important dystrophic proteins that would need to be confirmed with future experiments with additional animals and appropriate quantitative and statistical analysis. Many researchers have reported that sustained activation of the nuclear factor-kappa B (NFκB) pathway is involved in inflammatory myopathies and DMD. Thus, we next tested whether administration of bortezomib reduced NFκB levels. Based on 10 random micrographs per dog of muscle sections stained for phospho-NFκB (the active phosphorylated form of NFκB), there was a lower level of staining in the treated dogs compared to untreated dogs. This result indicates blockage of IκK and the consequent inflammatory process (Figure 6). Our immunohistochemical analysis revealed increased staining for phospho-NFκB in atrophic and regenerative fibers, and quantitative studies of positive nuclear activity showed significantly lower levels in treated dogs than untreated dogs (p<0.05) (Figure 6).

### Clinical pathology and clinical signs

Serum creatine kinase (CK) levels were dramatically elevated in both groups of GRMD dogs compared to healthy dogs at all time points, as were the levels of serum alanine aminotransferase (ALT) and aspartate aminotransferase (AST). The level of these enzymes peaked at the fourth week when the dogs were four months old, indicating muscle necrosis featuring dystrophic myopathy. We also observed a high serum electrolyte level (phosphorus, potassium) and a significant hematological finding of thrombocytosis. Urea and creatinine levels were within the laboratory reference ranges (Table 1).

**Table 1:** Serum and hematological parameters in GRMD dogs.

Dogs	Untreated dogs (CD)			Treated dogs (TD)		Refer. values
Parameter	K3	K4	K6	K1	B7	
**CK (UI/L)**	14436	15168	8512.6	15906	8779.3	**68–200**
**ALT (UI/L)**	503.1	420.3	304.3	405.5	311.2	**10–130**
**AST (UI/L)**	348.2	417.1	190.8	306.9	244.6	**23–66**
**Phosphorus (mg/dL)**	7.7	9	8.7	8.7	8.6	**2.5–6.2**
**Potassium (mEq/L)**	5.5	5.4	5.9	5.2	5.6	**3.6–5.8**
**Platelets (µL)**	521667	512667	717556	591000	561444	**2–5 10^5^**

Mean values for altered serum and hematological parameters from GRMD dogs during the nine weeks of the study. The times represent the bortezomib treatment period in the treated dogs (K1 and B7). The parameter with the greatest change was serum CK concentration, with a high magnitude range of 40 to 70 times the reference value from Kaneko et al. (1997). CK: creatine kinase

All of the GRMD dogs showed progressive clinical signs with muscle atrophy involving all four limbs, exercise intolerance, and abnormal gait. Dysphagia, regurgitation, and dyspnea may occur as a result of hypertrophy of the lingual, pharyngeal, and esophageal musculature. We observed different phenotypes in the GRMD dogs, even though they were the same age and four of them were littermates. Table 2 indicates the main clinical signs observed in all dogs, regardless of whether they were treated or untreated.

**Table 2:** Clinical signs in GRMD dogs.

Dogs age		
**4 months**	**5 months**	**6 months**
**- Rigidity of pelvic and thoracic limbs** **- Gait abnormalities**	-Clinical signs of 4 months plus:- Quadriceps rigidity- Hard to open mouth- Muscle atrophy	Clinical signs of 5 months plus:-Excess salivation and dysphagia-Increase in the resting respiratory rate and excessive abdominal component to breathing- Muscle contractures

The main clinical signs observed in GRMD dogs during treatment with bortezomib (three, four and five months of age). There were different phenotypes, but the main clinical signs related to muscular dystrophy progression were common to all of the dogs in the study. For physiological studies of GRMD, please see the reference [63] showing an *in situ* protocol to measure the force generated by a single muscle in dogs.

One treated dog (B7) presented bilateral acute conjunctivitis in the last treatment cycle (receiving 1.6 mg/m^2^ of bortezomib). The sclera of the dog was slightly reddish, with the periocular area crusty and lacerated. This dog was sent to the ophthalmology clinic for Small Animals at the University of São Paulo (FMVZ-USP) for additional exams. The dog was shown to be suffering from an acute allergic reaction, suggesting that the drug treatment could induce lesions. In another treated dog (K1), the progressive clinical signs of muscular dystrophy induced contractures and deformations in caudal limbs. These signs began at four months of age and continued until the dog became unable to walk with the caudal limbs. The untreated dog (K4) presented progressive clinical signs of muscular dystrophy beginning at 8 months and causing death at 10 months.

### Proteasome activities in blood lysates

Because chymotrypsin-like activity is the principal target of proteasome inhibitors, this activity was evaluated for each of the samples as a measure of proteasome activity. Effect of the drug is dose-dependent, with a positive change in fiber integrity only occurring at the maximum dosage of proteasomal inhibitor [27].

Twelve tests were conducted, with a minimum of three tests and a maximum of five tests from each animal per time point. Control samples were also used in all experiments. As expected, the negative control group of healthy animals had low chymotrypsin-like activity, while muscle extract from cachectic rats with high chymotrypsin-like activity was used as a positive control.

During the first cycle, with the dose set at 1.3 mg/m^2^, the treated GRMD dogs B7 showed and K1 showed 72.6% and 66.1% proteasome inhibition, respectively, during the first hour of evaluation. In the second cycle, a higher rate of inhibition was shown compared with the first cycle, with B7 demonstrating 80.6% proteasome inhibition. K1 showed a lower value of proteasome inhibition at approximately 47.6%.

**Figure 7:**
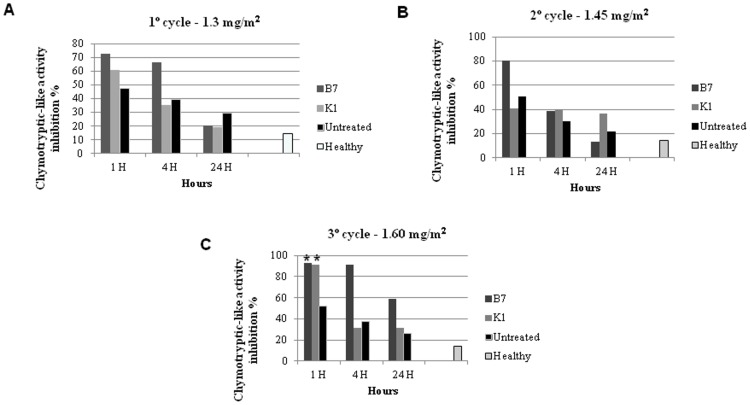
Chymotrypsin-like activity in blood lysate. Representative scheme for the *in vitro* assessment of 20S proteasome chymotrypsin-like activity by monitoring the release of the fluorophore AMC from the synthetic peptide substrate LLVY-AMC. We analyzed the chymotryptic-like activity in the blood lysate of TD, CD, healthy dogs (negative control) and the cachectic muscles taken from mice (positive control). Significant inhibition of 20S proteasome activity was observed within 1 hour of dosing in all cycles of treatment, after which the inhibition decayed. **A:** B7 and K1 showed higher proteasome inhibition at the first hour after treatment. **B:** B7 showed higher inhibition at the first hour after treatment. **C:** B7 and K1 showed higher proteasome inhibition at the first hour (** p<0.05) and four hours after treatment.

Maximal inhibition of the proteasome levels in blood was observed 1 hour after bortezomib administration in the last cycle when the dose was 1.6 mg/m^2^, and a satisfactory level of inhibition was maintained for 4 hours. Proteasome inhibition increased to 90% for dogs B7 and K1. After 24 hours, the levels of inhibition were similar to those of the CD (Figure 7). These data show that bortezomib inhibits 20S proteasome activity in the plasma of treated GRMD dogs in a dose-dependent manner, with maximal inhibition 1 hour after drug administration for each cycle studied.

In the third cycle, the last dose resulted in up to 90% inhibition of 20S proteasome activity, and dog B7 showed acute conjunctivitis and periocular lacerative lesions, side effects also found in 3–4% of human patients treated with bortezomib (Millennium Pharmaceuticals, unpublished data).

## Discussion

Recent studies have demonstrated that inhibitors of the proteasome pathway can effectively block the degradation of dystrophin and dystrophin-associated proteins and ameliorate the myopathic changes characteristic of dystrophin-deficient skeletal muscle [19], [27], [30] and α-sarcoglycan-deficient muscle [42].

We studied muscle fiber morphology at time 0 (before treatment) for both groups (TD and CD). The predominant histopathological signs were myofiber hypertrophy, fiber necrosis and regeneration, discrete endomysial fibrosis and marked myofiber atrophy [6], [43].

At the end of the treatment, treated dogs showed a more uniform fiber diameter and less lymphocytic invasion, suggesting that the inflammatory process was attenuated and that there was reduced connective tissue deposition in the endomysium and perimysium of muscle fibers. These results are relevant because the accumulation of endomysial and perimysial connective tissue in unused and dystrophin-deficient muscles may contribute to the impairment of intra-muscular blood circulation. The scarcity of capillaries may, in turn, lead to increased amounts of connective tissue, initiating a vicious cycle [33]. It has also been suggested that this scar tissue may itself assume a pathogenic role and contribute to disease progression by interfering with effective muscle regeneration and reinnervation [44].

One significant difference between the biopsies at T0 and T1 was the presence of inflammatory cells in the endomysium of the fibers in the T1 samples. Same observation was found at literature and suggested that mast cell degranulation plays a role in inducing myofiber death [45]. Mutations in dystrophin result in membrane damage, allowing massive infiltration of immune cells, chronic inflammation, necrosis, severe muscle degeneration [46] and an invasion of inflammatory cells [47] such as macrophages and T-lymphocytes [48].

Ultrastructural studies of Duchenne dystrophy indicate that hypercontraction of fibers is an early change, followed by myofibrillar lysis and total fiber necrosis, with macrophage invasion as a late event [49]. Although the integrity of the plasma membrane and mitochondria appeared to be preserved during the early stages of degeneration [50] and noted no structural abnormalities in adjacent normal-appearing fibers, we observed hypercontracted fibers in both groups. The most important sign of degeneration was highly electron-dense mitochondria, suggesting degeneration. We also noted macrophages inside the fibers, degenerated products, activated fibroblasts and connective tissue deposition in the endomysium and perimysium of the fibers[50].

Previous reports have shown that the effect of proteasomal inhibitors (MG-132, bortezomib and MLN-273) is dose-dependent and that positive changes in fiber integrity only occur at the maximum dosage delivered [19], [26]. In light of these reports, we chose to measure the percentage of proteasome inhibition in blood cells for each cycle of bortezomib treatment. The recent literature has shown a diversity of expression and activities for the ubiquitin-proteasome and calpain systems in GRMD muscle, leading to unexpected consequences such as heart-related cardiomyocyte lesions in response to pharmacological inhibition [51].

Previous *in vitro* studies have shown that maximal proteasome inhibition occurs 1 hour after administration of bortezomib [41], [52]. Reports [35] demonstrated maximal inhibition of the 20S proteasome, one hour after drug administration. The 20S proteasome activity began to recover after 4 hours, and 50% recovery had occurred at 24 hours. These studies agree with our present results.

In the final cycle, we observed up to 90% inhibition of proteasome activity in drug-treated dogs, but we observed an adverse effect, namely acute conjunctivitis, as well. The dose-limiting toxicity in animal species occurs at approximately 90% 20S proteasome inhibition. Although the specific major dose-limiting toxicity (DLT) varied from schedule to schedule, neuropathy, diarrhea, and fatigue are the predominant toxicities observed in treated humans [53]. Chronic proteasome inhibition is associated with increased coronary artery oxidative stress and early atherosclerosis [54]; because cardiomyopathy is a consequence of DMD, the chronic use of bortezomib should be associated with other therapies and used with caution in these patients. We observed no significant differences between the biochemical profiles of treated and untreated dogs. The most important findings were increases in the CK, AST and ALT levels in all GRMD dogs over time. The CK serum concentration is relevant for the diagnosis of muscle diseases, and muscular dystrophy in dogs is associated with increased CK [2], [3], [55]. AST and ALT are also involved in muscular dystrophy, and similarly to CK, their increased levels suggest muscle necrosis and myopathy [42], [56]. Our results indicated progression of muscle disease in both groups of dogs, even though we observed rescue of the DGC proteins and amelioration of the muscle fibers. Our study demonstrates for the first time not only the rescue of DGC proteins but also the clinical signs associated with the use of proteasome inhibitor in dogs with muscular dystrophy. To evaluate the possible rescue of dystrophin and dystrophin-associated proteins, we performed immunohistochemical analyses of dystrophin and α- and β-dystroglycan. We were unable to demonstrate dystrophin rescue in muscle membranes. However, drug-treated dogs showed increased α- and β-dystroglycan expression, indicating an improvement in the disease histopathology phenotype.

Previous reports have shown increased NFκB in muscle atrophy [57] and muscular dystrophy [17],[18], [58], [59], and it has been reported that proteasomal inhibitors are able to block the activation of NFκB in the muscle fibers of mdx mice [15].

By blocking proteasomal activity, bortezomib prevents the activation of NFκB, thus reducing the accumulation of anti-apoptotic factors and inflammatory molecules. We performed an immunohistochemical analysis for phospho-NFκB (the active form of NFκB) and observed increased levels of phospho-NFκB in untreated dogs. This result confirmed the morphology we observed, with more inflammatory and abnormal fibers in untreated dogs. Intense immunostaining for phospho-NFκB was observed in atrophic, necrotic and regenerating fibers and primarily appeared near areas of mononuclear cell infiltrates [26]. Similar results were found using the proteasome inhibitors bortezomib and MLN273 [19].

The activation of NFκB and the acute activation of TGF-β1 in human dystrophin-deficient muscle cause metabolic pathway failure later in the disease and appear to be associated with symptoms and muscle wasting in DMD [21]. Proteasome inhibitors may ameliorate the dystrophic process by preventing the proteolysis of the dystrophin complex, thereby preserving the stability of the muscle fiber and modulating the fibrogenic (TGF-β1) and inflammatory responses (NFκB) [60]. TGF-β1 release from muscle degeneration may trigger the activation of extracellular matrix proteins, and this activation could lead to connective tissue proliferation [21], [61]. Untreated dogs had higher levels of TGF-β1 expression as determined by immunohistochemical analysis, microscopy and ultrastructural analysis. This result suggests that treatment with bortezomib may decrease connective tissue formation and fibrosis in GRMD dogs.

The expression of TGF-β1 in the early stages of DMD may be critical for initiating muscle fibrosis, and antifibrosis treatment might slow the progression of the disease, increasing the utility of gene therapy [21], [22]. We started bortezomib treatments before moderate-to severe clinical signs appeared in these dogs so that the possible rescue of DGC proteins could be evaluated before the fibrotic process had fully developed. Thus, we used dogs less than 5 months of age; however, the effects of this drug in older dogs, when the disease is well established, are not clear. It is now suggested in humans that a combination therapy using different agents leads to a better quality of life in these patients and decreases the complications of their degenerative and progressive illness [62].

## Conclusions

In conclusion, bortezomib can be safely administered as an intravenous bolus without premedication, and it is very well tolerated in dogs when administered according to the dose and schedule recommended. Inhibition of the proteasome was dosage-dependent, with maximal proteasome inhibition in the plasma of treated dogs occurring at the maximum dosage during the last cycle of treatment. Bortezomib was able to reduce the infiltration of inflammatory cells into the muscle fibers of TD, and the dogs had a lower deposition of connective tissue as a result of necrosis and muscle atrophy than CD. This finding suggests that a proteasomal inhibitor can improve the histopathological appearance of dystrophin-deficient skeletal muscle. While dystrophin was not rescued, dystrophin-associated proteins belonging to the DGC complex (β-dystroglycan and α-dystroglycan) were rescued in treated dogs. Our findings suggest that bortezomib can block the activation of phospho-NFκB, which may be important in the pathogenesis of DMD and may represent one line of research in Duchenne therapy. No single treatment will be sufficient to reverse the signs of disease, and therapeutic combinations can provide opportunities to control and understand the disease condition. Unfortunately, other parameters, such as clinical signs and the serum biochemistry measurements of CK, AST and ALT, were altered in both groups studied, indicating that the disease progressed despite treatment.

## Supporting Information

Figure S1 Alpha-dystroglycan and GAPDH western blot analysis. **A:** nitrocellulose membranes were cut in two parts and exposed each one to alpha-DG or GAPDH. **B:** Alpha-dystroglycan analysis (clone IIH6, Santa Cruz Biotechnology) 1∶500 diluted in TBS. We observed 97 kDa band between 125 and 88 kDa in B7 (TD group) indicating rescue of alpha-dystroglycan after treatment with bortezomib. **C:** GAPDH analysis (clone 1D4 Gene Tex) 1∶1000 diluted in TBS, the 37 kDa band is observed between 36 and 72 kDa.
(TIF)

Figure S2 Beta-dystroglycan and beta-actin western blot analysis. **A:** nitrocellulose membranes were exposed to anti beta-DG and the antibody was then stripped, and the same membrane was revealed with anti beta-actin. **B:** Beta-dystroglycan analysis (clone 43DAG1/8D5, Novocastra) 1∶500 diluted in TBS. We observed 45 kDa band between 50 and 34 kDa, indicating rescue of beta-dystroglycan protein in TD group after treatment with bortezomib **C:** Beta-actin analysis (clone 8H10D10 Cell Signaling) 1∶10000 diluted in TBS, the 42 kDa band is observed between 50 and 34 kDa.
(TIF)
