# Supplementary figures and images for: Bortezomib (PS-341) Treatment Decreases Inflammation and Partially Rescues the Expression of the Dystrophin-Glycoprotein Complex in GRMD Dogs
Source: PLoS One. 2013 Apr 8;8(4):e61367. doi: 10.1371/journal.pone.0061367 (PMC3620287; doi:10.1371/journal.pone.0061367)

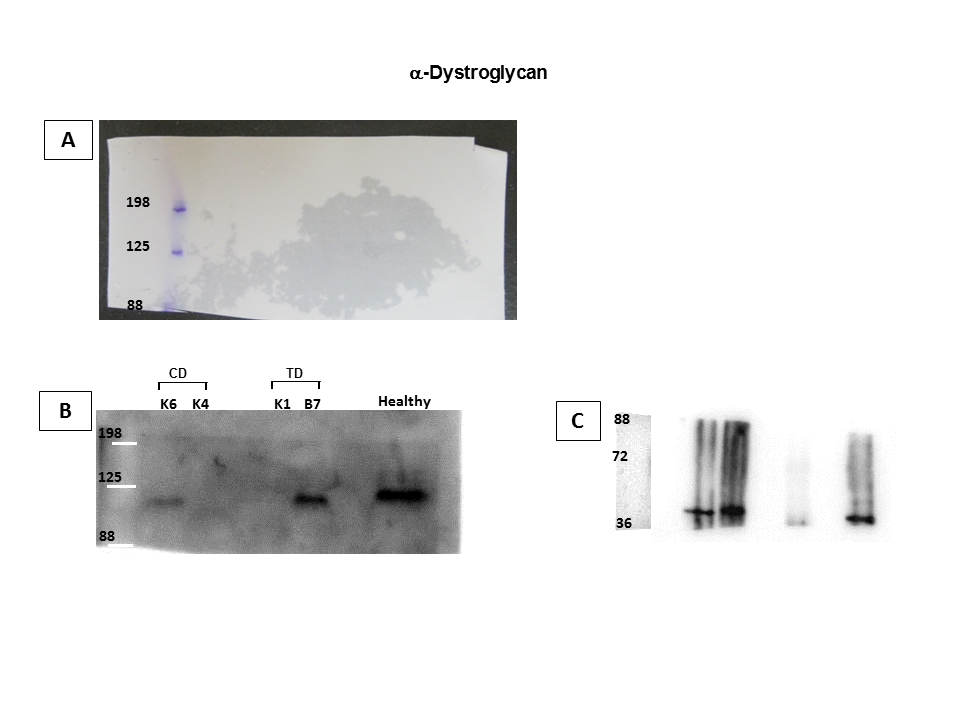

Supplement: Figure S1 — Alpha-dystroglycan and GAPDH western blot analysis. A: nitrocellulose membranes were cut in two parts and exposed each one to alpha-DG or GAPDH. B: Alpha-dystroglycan analysis (clone IIH6, Santa Cruz Biotechnology) 1∶500 diluted in TBS. We observed 97 kDa band between 125 and 88 kDa in B7 (TD group) indicating rescue of alpha-dystroglycan after treatment with bortezomib. C: GAPDH analysis (clone 1D4 Gene Tex) 1∶1000 diluted in TBS, the 37 kDa band is observed between 36 and 72 kDa. (TIF) [file pone.0061367.s001.tif]

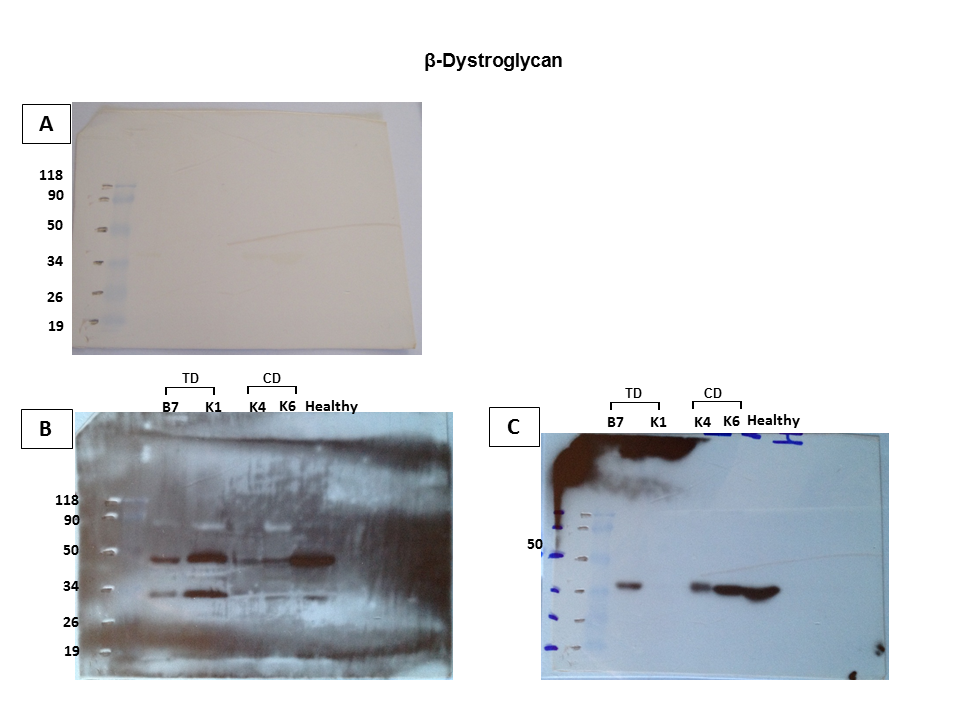

Supplement: Figure S2 — Beta-dystroglycan and beta-actin western blot analysis. A: nitrocellulose membranes were exposed to anti beta-DG and the antibody was then stripped, and the same membrane was revealed with anti beta-actin. B: Beta-dystroglycan analysis (clone 43DAG1/8D5, Novocastra) 1∶500 diluted in TBS. We observed 45 kDa band between 50 and 34 kDa, indicating rescue of beta-dystroglycan protein in TD group after treatment with bortezomib C: Beta-actin analysis (clone 8H10D10 Cell Signaling) 1∶10000 diluted in TBS, the 42 kDa band is observed between 50 and 34 kDa. (TIF) [file pone.0061367.s002.tif]
